# A Review of the Use of Microparticles for Cartilage Tissue Engineering

**DOI:** 10.3390/ijms221910292

**Published:** 2021-09-24

**Authors:** Rachel J. Kulchar, Bridget R. Denzer, Bharvi M. Chavre, Mina Takegami, Jennifer Patterson

**Affiliations:** 1 Department of Chemistry, Princeton University, Princeton, NJ 08544, USA; rkulchar@princeton.edu (R.J.K.); bchavre@princeton.edu (B.M.C.); 2 Department of Chemical and Biological Engineering, Princeton University, Princeton, NJ 08544, USA; denzer@princeton.edu; 3 Department of Molecular Biology, Princeton University, Princeton, NJ 08544, USA; takegami@princeton.edu; 4 Independent Consultant, 3000 Leuven, Belgium; 5 Biomaterials and Regenerative Medicine Group, IMDEA Materials Institute, 28906 Madrid, Spain

**Keywords:** regenerative medicine, mesenchymal stem cells, chondrocytes, hydrogels, drug delivery, cell expansion, bioreactors, bioprinting, microsphere, microcarrier

## Abstract

Tissue and organ failure has induced immense economic and healthcare concerns across the world. Tissue engineering is an interdisciplinary biomedical approach which aims to address the issues intrinsic to organ donation by providing an alternative strategy to tissue and organ transplantation. This review is specifically focused on cartilage tissue. Cartilage defects cannot readily regenerate, and thus research into tissue engineering approaches is relevant as a potential treatment option. Cells, scaffolds, and growth factors are three components that can be utilized to regenerate new tissue, and in particular recent advances in microparticle technology have excellent potential to revolutionize cartilage tissue regeneration. First, microspheres can be used for drug delivery by injecting them into the cartilage tissue or joint space to reduce pain and stimulate regeneration. They can also be used as controlled release systems within tissue engineering constructs. Additionally, microcarriers can act as a surface for stem cells or chondrocytes to adhere to and expand, generating large amounts of cells, which are necessary for clinically relevant cell therapies. Finally, a newer application of microparticles is to form them together into granular hydrogels to act as scaffolds for tissue engineering or to use in bioprinting. Tissue engineering has the potential to revolutionize the space of cartilage regeneration, but additional research is needed to allow for clinical translation. Microparticles are a key enabling technology in this regard.

## 1. Introduction

### 1.1. Tissue Engineering (TE)

In the United States (US) alone, there were upwards of 30,000 single-organ transplants, 2000 multiple-organ transplants, and 80,000 tissue transplants performed in the year 2020 [1]. Millions of Americans suffer from tissue loss or organ failure annually [2], meaning they require donated tissues and organs or assisted mechanical devices and artificial prostheses for treatment as the clinical standard of care [2,3,4,5,6,7]. In spite of these procedures benefiting countless lives, there exists a major donor shortage and limited availability of compatible donors [2,3,4,5]. As of February 2021, there were over 107,000 people on the US national transplant waiting list, but only 39,000 transplants were performed in 2020 [6]. This donor shortage only increases every year, growing the waiting list and resulting in more patient deaths [2]. Many organs from donors fail to be matched, transported, and transplanted into a patient within a suitable time frame [3]. Organs can also be discarded for transplantation if a facility deems that the donated organ’s inflammation, trauma, edema, or other conditions could potentially exacerbate the recipient’s preexisting health conditions [1]. Currently, three main alternative therapeutic mechanisms being researched to treat injured or diseased tissues are the implantation of cells grown under a controlled environment, the implantation of tissues developed in vitro from cells and scaffolds, or in situ tissue regeneration [4].

Tissue engineering (TE) is now being researched as an interdisciplinary biomedical engineering tool that addresses the complications found in organ donation as it can act as an alternative to tissue or organ transplantation [2,3,4,5]. TE technologies have the potential to fabricate healthy, bioengineered tissues (structural, metabolic, or both) for patients suffering from kidney, heart, liver, and other diseases and are being explored to treat bone defects, cartilage damage, pulmonary disease, and many others, ultimately having the potential to revolutionize clinical medicine while improving and saving hundreds of millions of lives [3]. After growth in vitro using a scaffold and patient or donor cells, the bioengineered, three-dimensional (3D) tissue can be implanted into the patient to retain, maintain, or improve the functionality of damaged tissues or whole organs [2,3,4,5]. In terms of cellular implantation, individual cells or a population of cells originating from either the patient or a donor, can be either directly injected into the damaged tissue (often termed cell therapy) or first combined in vitro with bioactive, degradable scaffolds [4]. The engineered tissues or organs have the potential to either be fully functional at the time of treatment or to create the functional tissue desired after implantation and further maturation in situ [5]. The success of TE relies on the interaction and integration of cells and tissues via appropriate cellular and physical signals [8]. More recently, researchers have attempted to use injectable materials, such as hydrogels (networks of crosslinked hydrophilic polymer chains which have the ability to absorb large amounts of water), to replace the need for invasive surgery [9,10]. The use of hydrogels in conjunction with microspheres has also been posed as a possible way to enhance the function and character of the transplanted cells [9,11]. These hydrogels support chondrocytes in maintaining their cellular phenotype [9]. Although TE has many hurdles to overcome, such as maintaining reliability and a low cost while meeting governmental and societal standards, its potential to transform the medical system by eliminating the need for tissue and organ donors continues to encourage further research and innovation [4].

### 1.2. Background on Microspheres, Microcarriers, and Granular Hydrogels

**Figure 1:**
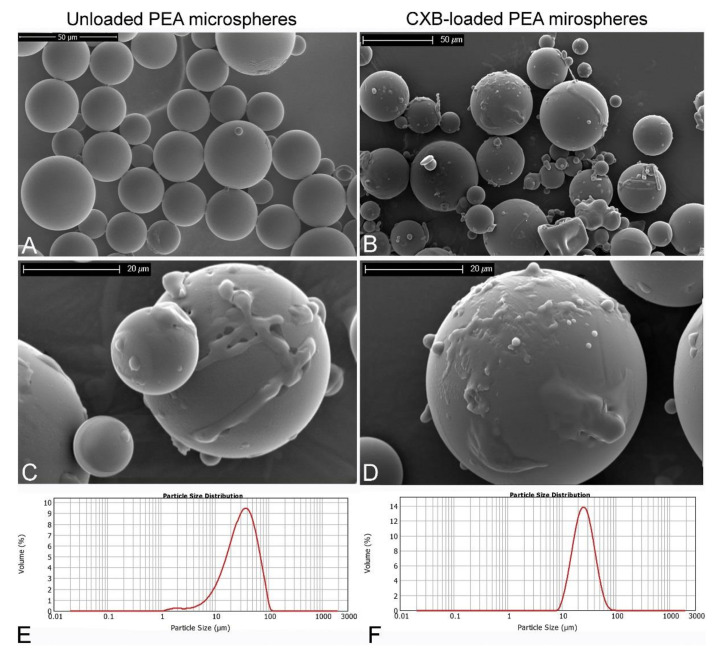
Scanning electron micrographs of polyesteramide (PEA) microspheres. (**A**,**C**) Control microspheres. (**B**,**D**) Microspheres loaded with celecoxib (CXB). (**E**,**F**) Respective particle size distributions. Scale bars are 50 µm in (**A**,**C**) and 20 µm in (**B**,**D**). Reproduced under the terms of a Creative Commons Attribution License from [18]. © 2021 The Authors. Published by Elsevier.

Microspheres are spherical particles with a diameter of 1 to 1000 µm (Figure 1) and are widely used in drug delivery due to their therapeutic advantages [12]. They are divided into two broad categories: microcapsules, in which an enclosed substance is surrounded by a distinct wall, and micromatrices, in which the enclosed substance is dispersed throughout the matrix [13]. Microspheres, which are particularly useful in drug delivery, should be able to incorporate high concentrations of drugs, remain stable after synthesis, release the active reagent with time control, and be susceptible to modification. As another application of spherical particles, microcarriers are essentially microspheres that are approximately 100–300 μm in diameter and typically used suspended in a growth medium. They act as support matrices and allow cells to adhere to their surfaces and to proliferate [14,15,16]. Originally, microcarriers were mostly made from synthetic polymeric materials such as polystyrene, but recently, more focus has been directed to natural polymers due to their inherent biocompatibility and cell recognition capabilities [14,15,16]. Typically used natural polymers include carbohydrates (starch and chitosan), proteins (gelatin and collagen), and chemically modified carbohydrates (poly(acryl) starch). Common synthetic polymers are divided into biodegradable (lactides and glycolides) and non-biodegradable (poly methyl methacrylate) polymers. Commercial examples of microcarriers include gelatin-based CultiSpher, collagen-based Cytodex, and cellulose-based Cytopore [14,15,17].

Each type of microsphere and/or microcarrier exhibits different properties, such as density, porosity, and size, and they can be classified based on these characteristics [14,15,16]. First and foremost is the size and morphology, which includes the microsphere diameter and porosity. Size determines the potential function of the microsphere, as very small microspheres are used in lateral flow tests, while microspheres ten times their size are used for cytometric assays. Next is composition, which directly affects density, refractive index, and autofluorescence, giving the microspheres certain advantages or disadvantages when working in different environments. Microspheres can also be distinguished by their surface chemistry, which are the reactive groups, level of functionalization, and charge on their surfaces. These are important because microspheres can be coated with molecules such as proteins or antibodies to optimize their activity and reduce any nonspecific interaction. The hydrophobicity or hydrophilicity of the material also affects properties such as nonspecific binding. The last category is special properties, such as the incorporation of visible dyes/fluorophores or iron oxide for super-paramagnetism to aid with visualizing the microspheres [12,19].

Granular hydrogels are the aggregate of unique, viscoelastic, and micrometer sized hydrogels which have the potential to revolutionize the TE space [20,21]. The way that these hydrogels are prepared means they can be made from different types of particles, such as spherical microspheres and elongated microparticles, called microstrands [20,21,22,23]. Although not much research investigating granular hydrogels specifically for cartilage regeneration has been conducted, scaffolds made from granular hydrogels are a promising technique for future TE applications. Overall, microparticles are particularly interesting to consider in the context of TE approaches because they fall in a useful size range between macroscale biomaterial scaffolds and nanomaterials. They are small enough to be delivered in a minimally invasive way through a syringe and needle, such as with the microspheres used for controlled release of drugs, but they are also large enough to provide a surface for cells to grow on, particularly in the case of microcarriers, or to build up 3D structures in the case of granular hydrogels. In the context of cartilage TE, the use of biomaterial scaffolds, including those based on hydrogels [24,25,26], as well as the use of nanomaterials, particularly nanoparticles used for controlled delivery [27,28], have been extensively and recently reviewed elsewhere.

### 1.3. Overview of the Review

This review investigates the versatile applications of microparticles, including microspheres, microcarriers, and granular hydrogels, to cartilage TE by first providing background into TE and cartilage regeneration, then transitioning into a review of microspheres’ ability to act as drug delivery vehicles for targeted injection into tissue for pain reduction or stimulation of cartilage regeneration. Microspheres can also be used for engineering cartilage tissue constructs in vitro with potential application to be implanted in vivo as parts of cell sheets or engineered tissues. In the following section, we discuss how microcarriers can serve as surfaces for stem cells or chondrocytes to adhere to and proliferate on, allowing for large scale cell expansion, which is necessary for clinical TE and regenerative medicine therapies. Drug delivery and cell expansion are traditional applications of microspheres and microcarriers, respectively. This review finally also investigates newer techniques such as granular hydrogel scaffolds that have potential in 3D bioprinting of tissues and microporous annealed particle (MAP) scaffolds that can aid in stem cell implantation into tissues. Thus, we include both traditional and up-and-coming applications of microspheres and microcarriers to provide a comprehensive review of previous literature while including newer technologies to help aid in future experiments and clinical research for cartilage TE.

## 2. Cartilage Regeneration and Tissue Engineering (TE)

### 2.1. Cartilage Tissue and Disease and Current Treatment Options

Cartilage tissue is found in several locations within the body. Articular cartilage is a smooth tissue that displays both elastic and viscous behavior when deformed, has limited regenerative capabilities, and distributes weight across diarthrodial joints, the most mobile type of joint [29]. It also has unique tribological properties, such as having a low coefficient of friction and exhibiting a mixture of boundary and fluid film lubrication mechanisms [30,31]. The synovial fluid, which fills the joint space and is composed of molecules including glycosaminoglycans, the protein lubricin, and phospholipids, also contributes to the lubrication of the joints [30,32]. Annually, there are more than 1 million surgical procedures involving cartilage replacement in the US [2]. Moreover, osteoarthritis (OA), a degenerative disease that continuously wears down articular cartilage and causes extreme amounts of duress, affects around 27 million adults in the US, leads to over 50% of all joint replacement, and costs over $15 billion every year [33,34]. The intervertebral disc (IVD) is a cartilaginous structure consisting of fibrocartilage and links vertebral bodies together [35]. IVD degeneration typically occurs earlier compared to the degeneration of other connective tissue in the body, resulting in back pain. The prevalence of disc degeneration increases with age, affecting 40% of people aged 40 and about 80% of people of at least 80 years of age [36].

**Figure 2:**
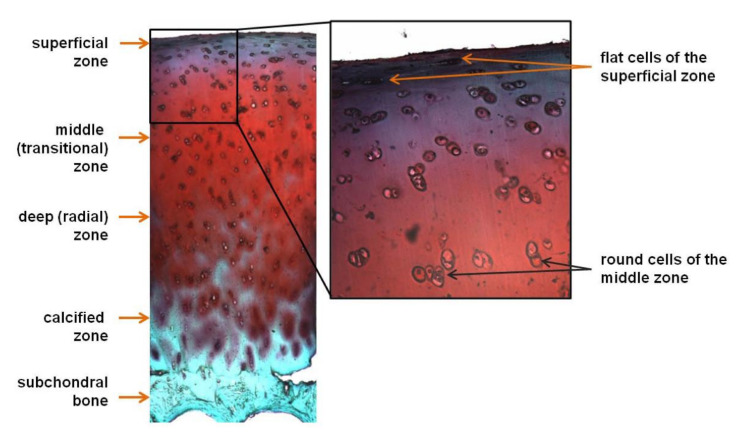
Histology images showing the structure of the different zones within a cross-section of adult human articular cartilage, stained with Safranin-O and Fast Green, and the corresponding morphologies of chondrocytes within the tissue. Reproduced under the terms of a Creative Commons Attribution License from [39]. © 2021 The Authors. Published by MDPI.

Chondrocytes, the primary cell type present in cartilage (Figure 2), are a homogeneous type of cell that facilitate extracellular matrix (ECM) turnover, largely regulate tissue homeostasis, and are relatively limited in number [29,33,34,37]. Thus, the cartilage damage caused by aging, disease, injury, or overuse may be irreversible and permanent [9,33,37]. Chondrocyte imbalance may result in degenerative diseases, with cartilage degeneration occurring as a direct result of the enlargement of chondrocyte cells and the expression of proteolytic enzymes [29]. The size of the chondral lesion influences the desired treatment approach. Smaller chondral lesions (<2–4 mm) can be treated with palliative treatment that involves the removal of damaged cartilage and the washing out of the defect with aqueous solution, treatment aimed at repairing the diseased area via marrow stimulation techniques, and restorative treatments, encouraging the utilization of microspheres [33,38]. Larger chondral lesions can be treated via the use of osteochondral allografting (partial replacement) or by entirely replacing the joint, encouraging cartilage TE applications [33]. However, surgical reconstruction rarely results in the full functional restoration of the cartilage to its natural state. Moreover, surgical cartilage treatments, including autologous chondrocyte implantation (ACI), often result in the formation of fibrocartilage, which is weaker and more transient than articular cartilage; the scar tissue of fibrocartilage has a higher coefficient of friction than articular cartilage, meaning that degeneration coupled with a limited range of motion is a likely outcome [9].

### 2.2. Cartilage TE Specifics

TE remains an area of immense opportunity as it has the potential to form cartilage via in vitro construction and implantation [9,34]. The cell, scaffold, and growth factors, although not always simultaneously used, are the three elements involved in cartilage TE approaches to regenerate new tissue [9,40]. When choosing cells for cartilage TE, it is essential to determine if the cells can be properly isolated from their place of origin and to ensure that their in vitro expansion will not permanently change their function and phenotype [8,41]. Although the utilization of some types of stem cells raises ethical concerns, they have the potential for application to cartilage TE and regenerative medicine because of their ability to differentiate into other types of cells and control anti-inflammatory and immune responses [14,42,43,44,45]. One of the main types of stem cells being studied for cartilage TE is mesenchymal stem cells (MSCs), and these are adult stem cells that can differentiate into different types of tissue such as cartilage and bone cells. Through the combination of cells with biomaterial scaffolds, researchers have been able to repair and create entirely new and healthy tissues [3,41]. During scaffold degradation, new tissue regeneration occurs, sometimes consuming an identical size and shape as the original scaffold [34]. Scaffolds in cartilage TE tend to be 3D, porous, and interconnected materials or hydrogels, and they are chosen based on their mechanical strength, biodegradability, biocompatibility, aptitude to facilitate cellular activities, flexibility to be oriented into different shapes and sizes, surface chemistry, etc. [8,9,34,46,47,48]. After being seeded with an appropriate cell source and/or filled with bioactive molecules, the combination of the scaffold and growth factors can provide structural support, promote cellular differentiation, and transmit cells which aid in tissue growth [8,9]. Proteins, carbohydrates, synthetic polymers, and composite materials commonly compose scaffolding material, and scaffolds can be woven, spun into nanofiber materials, or made into hydrogels [9]. There are two main ways in which the scaffold can be used. First, when the scaffold is only combined with growth factors or other bioactive molecules, cells can be recruited in vivo and migrate to the scaffold site to develop tissue throughout the matrix [8,41]. Alternatively, the scaffold can be pre-seeded with cells to create a tissue for transplantation. Moreover, recent attempts of utilizing bioreactors to provide mechanical loading and delivery of nutrients have been used in the preparation of bioengineered cartilage in vitro. Thus, the cell, scaffold, and growth factors all offer unique mechanisms when used in TE, with TE having the potential to help millions of people suffering from damaged cartilage. However, successful attempts to create 3D, bioengineered cartilage have also faced challenges. One area which poses limitations to the progression of utilizing TE for cartilage regeneration lies in the difficulty of engineering a weight-bearing tissue that is also made of a multiphasic cellular structure [9].

## 3. Microspheres as Delivery Vehicles for Growth Factors and Drugs

Microspheres can be used as delivery vehicles for growth factors and drugs when preparing constructs for TE. In approaches for cartilage regeneration, microspheres on their own have also proven to be a useful method of drug delivery due to their ability to be injected because of their small size and spherical shape [49]. Drug release rates can be regulated by modifying microsphere size and composition, allowing the treatment to be constant and prolonged. For example, the drug release rate decreased, and duration increased, with increasing monodisperse polylactic-co-glycolic acid (PLGA) microsphere diameter [50]. Microspheres can also help increase patient compliance because they provide an alternative to oral treatments that rely on patient action, as microspheres can be injected and locally release necessary drugs at a constant rate without additional interference. With this controlled release, the toxic side effects of drugs can be curbed, as seen in the use of PLGA microspheres, which decreased the systemic toxicity of the potent cancer treatment plumbagin in a murine tumor model [51]. Microspheres can further protect encapsulated cargo from degradation by enzymatic activity inside the body, such as from gastrointestinal fluids. Despite these benefits, microspheres also come with several limitations. When designing and developing new microsphere formulations, it is hard to reproduce and predict the full effects of the human body environment on injectable microspheres, including changes in pH, temperature, and enzymes [19,52,53]. Further, release rates from microspheres are rarely constant in practice and may suffer from a burst release or from a release profile that varies with time. Despite these limitations, microspheres have been researched as an approach to treat cartilage defects. With targeted treatment through injection into the joint spaces between cartilage or directly into the cartilage tissue itself, microspheres can be used to release drugs locally to reduce pain and stimulate regeneration of cartilage [54,55,56]. Different drugs researched for this application include corticosteroids, anti-inflammatories, and growth factors, which are discussed in depth throughout this section.

### 3.1. Corticosteroids

Corticosteroids, fast-acting steroid hormones, have commonly been used to treat symptoms associated with OA and joint inflammation since the 1950s [57]. Corticosteroids such as triamcinolone acetonide (TAA) are most effective when directly delivered to the site of inflammation, but are not efficient alone due to their short lifetime in the body. Therefore, microspheres can be used to extend corticosteroids’ therapeutic effects to treat symptoms of joint inflammation, particularly cartilaginous pain [58]. However, corticosteroids must be administered in moderation, as long-term exposure can result in adverse effects [59]. Rudnik-Jansen et al. analyzed the controlled release of TAA, using polyesteramide (PEA) microspheres to prolong pain relief and to avoid negative side effects for IVD degeneration, and they tested this in a large animal model in canines [60]. The sustained release of TAA did not affect disc height index in the induced IVD degeneration in vivo no matter what the concentration of TAA was in the microspheres. However, with low dosages of TAA in the microspheres, lower levels of nerve growth factor were detected in the surrounding nucleus pulposus (NP) tissue. Because increased nerve growth factor levels are associated with inflammation and chronic lower back pain, the results indicated that extended release of TAA from the microspheres was likely to reduce pain from IVD degeneration [60]. In another study, Kraus et al. compared the effects of TAA delivered with a prolonged release, by encapsulating it in PLGA microspheres, or with a rapid absorption, using a crystalline suspension of the drug [61]. Results from this Phase 2 clinical trial showed that microsphere delivery lengthened the amount of time TAA was present in the synovial fluid. With the microspheres, TAA was quantifiable through 12 weeks after treatment, while only two of eight patients had quantifiable synovial fluid TAA at 6 weeks for the crystalline suspension. In addition to the longer time of localized exposure, the use of microspheres decreased systemic TAA exposure compared to the suspension, making the microspheres a more desirable candidate to relieve OA pain [61]. This application of microspheres with TAA has been approved by the Food and Drug Administration under the name of Zilretta (FX006) and is currently the only approved treatment for treating OA knee pain through intra-articular use [62,63,64].

Another synthetic corticosteroid is dexamethasone, which is usually used to relieve inflammation for musculoskeletal diseases [65]. It has been formulated into microspheres to provide extended release and to locally reduce inflammation [66]. Further, microspheres releasing dexamethasone can be used as a supplement for chondrogenesis and maintenance of mature tissues. For example, dexamethasone was delivered via microspheres that were made from PLGA and designed to release the drug for over 99 days. After in vivo experimentation in adult mongrel dogs using an osteochondral autograft model, dogs treated with the dexamethasone-loaded microspheres (DLMS) were almost three times more likely to have better chondrocyte and proteoglycan Osteoarthritis Research Society International (OARSI) scores measuring OA progression compared to dogs treated without dexamethasone (control) and dogs injected with the dexamethasone directly (INJ). Staining with toluidine blue indicated better proteoglycan deposition, indicative of the formation of cartilage ECM, in the DLMS group, and collagen sub-scores also were much better in the DLMS group than in the control and INJ groups. These histological findings were supported by the gait of the dogs—the gait of control and INJ groups were worse than the established baseline six months into the experiment, but DLMS gait remained similar to the baseline. Overall, this experiment supported the use of dexamethasone in microspheres to improve osteochondral graft outcomes [67].

### 3.2. Anti-Inflammatories

**Figure 3:**
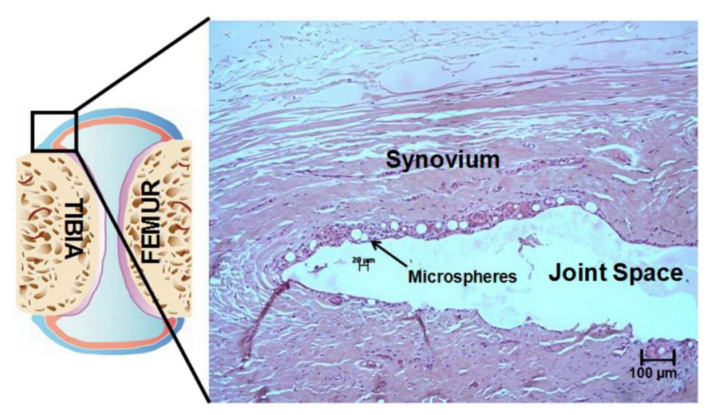
Histology image of a cross-section of the joint space and surrounding tissues, stained with hematoxylin and eosin, showing microspheres entrapped in the synovial tissue three weeks after injection into a healthy rat knee. Scale bar is 100 µm. Reproduced with permission from [69]. © 2021 Elsevier.

Because cartilage impairment is often associated with inflammation, treatments with anti-inflammatories, many of which are pain relievers, are an effective method to prevent and treat cartilage degeneration and OA [68]. However, treating the inflammation and/or pain alone can have long term consequences. Non-steroidal anti-inflammatories (NSAIDs), which are common, over-the-counter anti-inflammatory medicines, are good for treating inflammation and providing pain relief, but with the possible gastrointestinal and cardiac side effects that may come with extended use, they are not a suitable treatment for chronic pain. In addition to pain management, celecoxib has been used to treat cartilage degeneration due to its protective effect on osteoarthritic cartilage and ability to relieve OA pain. Celecoxib, an anti-inflammatory and analgesic used for treating OA, was formulated into PEA microspheres and tested in vitro and in vivo for its effect on the inflammatory response, biocompatibility, and degradation in a study performed by Janssen et al. [69]. Microsphere injections to treat rat knees in an OA model revealed celecoxib was released from the microspheres and remained bioactive for throughout 80 days in vivo. Moreover, histological assessment showed that the microspheres were found in the synovium with no necrosis or any other negative reaction to the injection but were encompassed by mononuclear inflammatory cells and giant cells without endocytosis (Figure 3), consistent with the ability of the synovial membrane to remove foreign particles [69]. Oral delivery of cyclo-oxygenase-2 (COX-2) inhibitor drugs such as celecoxib is known to work but to have side effects including abdominal pain, nausea, and insomnia, so using microspheres to deliver them is also a potential pathway for improving treatment of IVD degeneration. Tellegen et al. tested celecoxib in vivo in a canine IVD degeneration model using the PEA microspheres shown in Figure 1 as a delivery system. A 28-day extended release, inflammation inhibition, and anti-catabolic effects in NP cells were observed. Histological scores showed that, in the in vivo experiment, treatment with low dose and high dose celecoxib microspheres resulted in lower synovial inflammation than treatment with unloaded microspheres, indicating disc degeneration inhibition [18].

Anti-inflammatory cytokines, another group of anti-inflammatory molecules, can promote cartilage recovery by suppressing inflammation and delaying joint space narrowing, and these have also been formulated into microspheres for controlled release. For example, cationic cytokines interleukin (IL)-4, IL-10, and IL-13 were delivered in gelatin microspheres composed of a crosslinked gelatin–genipin matrix whose degradation rate was dependent on the collagenase concentration in the surrounding environment. The release of the cytokines directly correlated with the degradation of the matrix, and the crosslinking was engineered to ensure that degradation would happen in response to the proteolytic enzymes present during an inflammatory flare. IL-4 and IL-13 revealed promising results in vitro as there was a decrease in nitric oxide production (an OA phenotype) within three days of them being delivered via microspheres into cell cultures composed of a mouse chondrogenic line and a primary human lung fibroblast line. There was no indication of adverse effects of the degraded microspheres on the surrounding cells, which was promising as well [70]. Further experimentation will be required to test the safety and efficacy in vivo, but these anti-inflammatory cytokines have great potential for treating OA when combined with microspheres.

The drug resveratrol inhibits IL-1β, which is a pro-inflammatory cytokine that activates matrix metalloproteinase 13, an enzyme that destroys cartilage matrices, and thus it is an interesting and potentially anti-inflammatory molecule to consider for treatment of cartilage degeneration. To this end, resveratrol was loaded into PLGA microspheres for sustained release, and its effects on human bone marrow-derived MSCs were observed. Results indicated that the released resveratrol was able to inhibit IL-1β and upregulate characteristic chondrogenic markers such as Col2, aggrecan, and Sox9 [71]. For future experiments, this response in inflammatory conditions will have to be researched further to see if these effects are enough to promote cartilage regeneration.

A final group of anti-inflammatory drugs commonly researched for cartilage treatment include statins. Statins, a cholesterol-lowering medication, come in several forms including atorvastatin, fluvastatin, and lovastatin. Fluvastatin, known for its anabolic and anti-catabolic effects on human OA chondrocytes, was studied as a potential candidate for OA treatment and formulated into microspheres for controlled release. During in vitro characterization experiments, the PLGA microspheres released fluvastatin in two stages, first as a burst release of about 70% in the first three days and second as a gradual release for the remaining two weeks where morphological changes occurred. Then, after five weeks of an experiment conducted in vivo in osteoarthritic rabbits, histological evaluation showed that all groups but the fluvastatin loaded microsphere group experienced OA progression. The fluvastatin loaded microsphere group saw no proteoglycan loss and had a much lower OARSI score compared to the other groups. Due to the short length of the experiment, the effects of fluvastatin could only be understood for early-stage OA. For further testing, using a larger animal model is necessary to be able to obtain synovial fluid as a benchmark, and a longer experimentation period is necessary to see the effects on later OA stages [72].

### 3.3. Melatonin

Melatonin is another potentially useful bioactive molecule for cartilage regeneration, due to its ability to promote chondrogenic differentiation of MSCs [73]. Therefore, poly(N-isopropylacrylamide)/hyaluronic acid hydrogels with PLGA microspheres containing melatonin were tested in a study conducted by Atoufi et al. The PLGA microspheres were coated with chitosan-g-acrylic acid containing allyl groups to act as crosslinkers for the hydrogels and aided in improving mechanical properties of the hydrogel so that it could better mimic cartilage tissue. The hydrogel integrated well with the natural cartilage, which was derived from cow ribs *ex vivo*, and the presence of melatonin was shown to increase glycosaminoglycan synthesis and to promote MSC differentiation when the cells were encapsulated in the hydrogels. Melatonin, a relatively small molecule, was gradually released from the microspheres through diffusion and degradation [74]. In an alternative approach, Naghizadeh et al. covalently bound melatonin to microspheres made of chitosan prior to including them in a hydrogel made of alginate to provide extended release, which led to increased cartilaginous matrix deposition by MSCs in vitro [75]. Further research on the delivery of melatonin from microspheres is warranted, as it has showed pro-chondrogenic effects when formulated into electrospun polycaprolactone fibrous membranes [76] as well as into forsterite nanoparticles that were embedded in a gellan gum and lignocellulose nanofibril composite [77]. It would also be interesting to explore alternative sources such as plant-derived melatonin, called phytomelatonin [78]. Ultimately, additional testing is necessary, especially in vivo testing, to consider melatonin as a possible drug to promote cartilage tissue regeneration.

### 3.4. Gene Therapy

Gene therapy involving viral vectors can be used to target nucleic acids for cartilage repair [79], focused on the idea that increasing the expression of certain genes can promote cartilage regeneration [80]. The delivery of plasmid deoxyribonucleic acid (pDNA) in gene therapy approaches to treat cartilage degeneration can be facilitated by microspheres. In a recent study conducted by Feng et al., pDNA and a hyperbranched polymer were self-assembled into polyplexes that could transfect the pDNA into NP cells. The polyplexes were encapsulated into PLGA microspheres to temporally control their release and ensure high delivery efficiency. They were then co-injected with spongy microspheres to concentrate the cellular transfection of the pDNA into the NP of a rat tail. This two-step delivery system was tested in vitro for the ability to repress fibrosis in NP cells. The particular pDNA coded for a nuclear receptor 4A1, which when inactive, leads to lasting activation of transforming growth factor (TGF)-β signaling and pathological tissue fibrosis. Therefore, by delivering 4A1 pDNA for constant activation, fibrosis associated NP degeneration could be stopped. Results of the in vitro experiment showed that the amount of stress fibers was much less with the 4A1 pDNA group than with the control group, which had blank pDNA. In vivo testing for treatment of IVD degeneration in rat tails revealed similar results. In groups without treatment (blank pDNA), there was almost no disc height and severe fibrosis in the center of the NP tissue, whereas the group that received the 4A1 pDNA treatment saw an increase in translucent disc matrix and a decrease in fibrous tissue. However, there was a narrowing disc height index (indicated by the IVD height) and fibrous tissue invasion based on appearance and microcomputed tomography (µCT) images. Histological assessments were also done, and the treated area in the group that received the pDNA was similar to the control (no fibrosis and no treatment) and had high glycosaminoglycan content [81].

### 3.5. Decellularized Cartilage Matrix (dCM)

There are several treatment options to address the progressive degradation of articular cartilage including microfracture and ACI, but these methods cannot regenerate functional cartilage. In an effort to treat osteochondral injuries with a focus on cartilage regeneration, decellularized cartilage matrix (dCM), with most of its proteins, cytokines, and growth factors still intact, is an interesting biological material to explore. Decellularization is a process that allows for the removal of genetic material while preserving structural and biochemical integrity, which allows for recellularization to produce more tissue [82]. This technology has potential application for growing cell sheets or engineered tissues in vitro, then implanting them as cartilage tissue structures into a patient. In a study by Ghosh et al., dCM was incorporated into polylactic acid (PLA) microspheres, and the microspheres’ ability to release dCM was tested in vitro with human MSCs (hMSCs). The contents were released over the course of four weeks, and the researchers determined that there was an increase in chondrogenesis. Viable cells were discovered on the surface of the microspheres seven days after the culture began, and they tended to attach and grow in slits between the microspheres. Cells grew on all groups in the experiment, but the number of viable cells was much higher on microspheres with the dCM compared to microspheres without the dCM. Cells in the dCM group portrayed more prominent sulfated glycosaminoglycan staining, and they were organized in a high-density, compact structure [83]. This method could be a potential treatment for cartilage regeneration, but more testing in vivo is needed to demonstrate safety and efficacy. Long term tests would also be desirable to see whether the newly differentiated cells would survive and replicate.

### 3.6. Growth Factors

**Figure 4:**
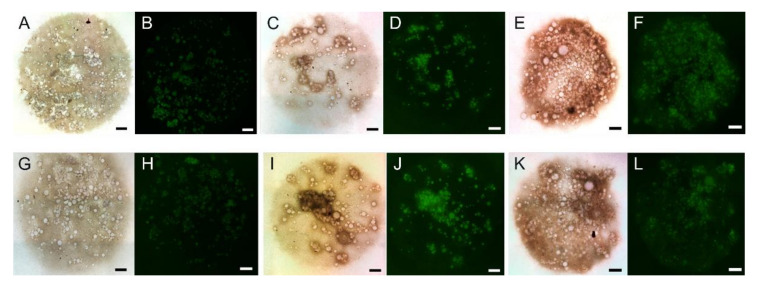
Brightfield and fluorescence microscopy images of micromasses of human periosteum-derived cells (hPDCs) combined with genipin crosslinked gelatin microspheres, which appear as fluorescent green spheres, with or without transforming growth factor (TGF)-β1. (**A**–**F**) After 2 weeks of in vitro culture. (**G**–**L**) After 4 weeks of in vitro culture. Scale bars are 500 µm. Reproduced with permission from [85]. © 2021 Elsevier.

Growth factors are natural proteins that can be used to stimulate cellular growth [84], and several of them can be applied in cartilage regeneration and TE approaches due to their ability to regulate development of articular cartilage and chondrogenic differentiation. Thus, they are also interesting to consider for controlled delivery from microspheres. Gelatin microspheres have been used as a method of releasing TGF-β1, which is a secreted protein involved in cell growth, cell proliferation, and cell differentiation [85,86]. These microspheres have been incorporated in a TE approach to create 3D pieces of cartilage tissue from stem cells in vitro by forming micromasses with the intention to later implant these in vivo. In a study by Kudva et al., TGF-β1 delivered via microspheres was found to promote the same level of chondrogenic differentiation in micromasses of human periosteum derived cells (hPDCs) compared to soluble TGF-β1 in the culture medium (Figure 4), indicating microspheres can be a viable alternative in this case [85]. Similarly, Solorio et al. used gelatin microspheres as part of self-assembled hMSC sheets to induce chondrogenesis. They showed that cartilage sheet thickness increased significantly in samples with the gelatin microspheres, whether TGF-β1 was delivered exogenously in the medium or was released from the incorporated microspheres [86].

From the same family as TGF-β1, the growth factor TGF-β3 has also been explored for cartilage regeneration. Sun et al. studied the use of PLGA microspheres to deliver TGF-β3 as a way to improve cartilage regeneration when combined with stem cell therapy. This in vivo experiment was conducted on rabbits with OA, separated into three groups: a control group injected with normal saline, one group injected with only human adipose derived stems cells (hADSCs), and one group injected with both hADSCs and TGF-β3-containing microspheres that had a sustained release of TGF-β3 over 30 days. All three groups of rabbits displayed different degrees of OA, with the control group exhibiting the most degeneration and the group with the hADSCs/TGF microspheres displaying the most articular cartilage regeneration [87]. In a different study, Morille et al. also demonstrated chondrogenic differentiation from stem cells by seeding MSCs in vitro onto PLGA-P188-PLGA-based microcarriers with a TGF-β3 coating. The researchers further investigated chondrogenesis in situ in mouse knee joints with OA by injecting the microcarriers seeded with MSCs, showing that cartilage tissue grew in the joints that contained the microcarriers with the TGF-β3 but not in those without TGF-β3 [88].

Other growth factors have been utilized in similar degradable microspheres to stimulate chondrocyte development. In a study by Vayas et al., PLGA scaffolds were prepared for cartilage repair with three layers: the first base layer with PLGA microspheres, the second layer with a suspension of PLGA microspheres encapsulating bone morphogenetic protein (BMP)-2, and the third layer with an electrospun PLGA thin membrane that included bone marrow-derived MSCs (bMSCs). Rabbits with a critical size osteochondral defect treated with scaffolds with only BMP-2 and with the bMSC-BMP-2 combination had a higher repair area, showing that this tri-layer scaffold was effective [89]. As a final example, Gavenis et al. used nude mice as a model to examine the efficacy of BMP-7 release from PLGA microspheres in vivo. The microspheres were added to a collagen hydrogel containing human osteoarthritic chondrocytes, and mice treated with the growth factor showed increased collagen Type II production compared to the control, showing that BMP-7 can be useful in cartilage formation [90].

In conclusion, microspheres have been used as drug delivery vehicles to address cartilage degeneration. While many drugs to treat cartilage degeneration in diseases are still under research and development, drug delivery using microspheres has been shown to be a viable alternative to current treatments because of their ability to provide sustained and localized release. Of them, Zilretta, an extended release TAA injection, has already been approved by the FDA to treat OA [62]. Other promising treatments include anti-inflammatories, gene therapy, and growth factors, which can be targeted to the necessary area using microspheres.

## 4. Microcarriers for Cell Expansion

Cell expansion is vital because it results in an increase in the number of cells often from millions to billions or even trillions, which is needed for effective regenerative medicine therapies [14,17,91]. It is also important because it addresses the limited number of primary cells available from a biopsy. Stem cells, such as MSCs, can be expanded with the use of cell culture flasks in vitro for small-scale experiments, but other techniques such as microspheres (also referred to as microcarriers in this context) in bioreactor systems are necessary for large-scale or clinical applications [14,15,17,44,45,92]. For cell expansion, it is vital that the medium allows for cell growth via exchange of nutrients and that there is enough surface area for cell adhesion and replication [14,93]. Microcarriers, especially those that are porous, offer the advantage of having a large surface area, thus allowing for large-scale cell expansion [15].

### 4.1. Microcarrier Fundamentals

**Figure 5:**
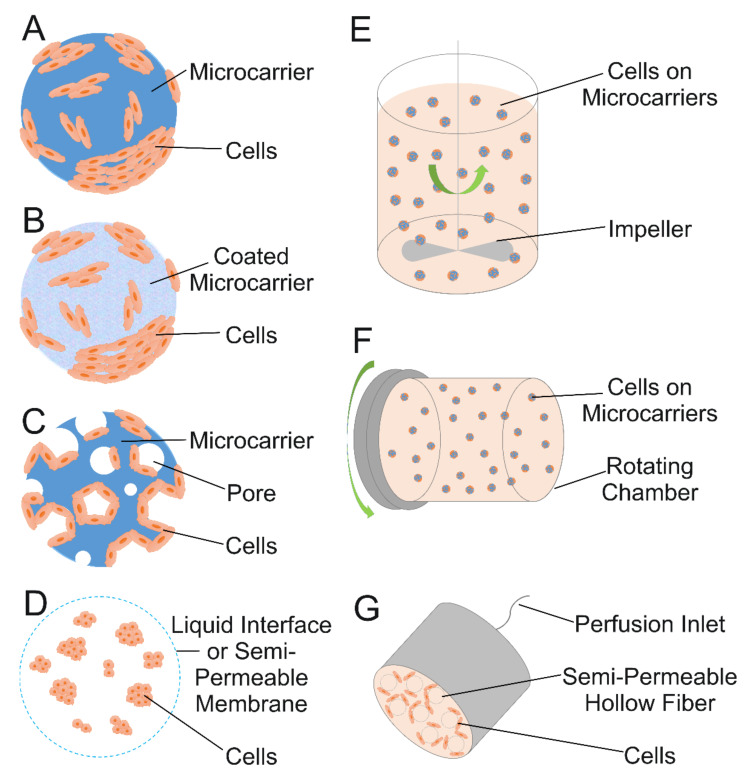
Schematics showing culture of cells on microspheres and in bioreactors. (**A**) Solid microcarrier with cells growing on the surface. (**B**) Microcarrier coated to promote cell attachment. (**C**) Porous microcarrier, with cells growing in pores. (**D**) Cells encapsulated in liquid microspheres. (**E**) Stirred-tank bioreactor. (**F**) Rotating flask bioreactor. (**G**) Perfused hollow fiber bioreactor. Schematics based on [104].

Microcarriers can be made from either natural or synthetic materials. The synthetic polymer microcarriers often have a positive surface charge in order to facilitate attachment of negatively charged cells, although this can make it more difficult to harvest the cells from the microcarriers [14,16]. To combat the difficulties regarding cell harvesting, proteolytic enzymes such as trypsin have been used to separate the cells from the microcarriers [14,15,17,94]. However, these enzymes can damage cell membrane proteins, resulting in a lower efficiency for TE and transplantation, making it more desirable to use microcarriers that do not require enzymes for cell harvesting, such as thermo-responsive microcarriers that allow the cells to detach when exposed to certain temperatures, as reviewed by Zhang et al. [94]. Other stimuli-responsive microcarriers exist, and they react to changes in environmental conditions, such as pH, temperature, light, stress forces, and biochemical substances [14,94]. Additionally, microcarriers can either be solid (with or without additional coatings), porous, or liquid (Figure 5A–D), with porous microcarriers containing pores that either solely reside on the surface or are deep enough to create internal channels for cells to grow in [14,15,16,17]. For solid and porous microcarriers, it is easier for cells to adhere to the solid surfaces and proliferate either on the surface or in the pores, but for liquid microcarriers that act in a liquid–liquid system, the cells reside at the interface of the immiscible liquids, such as that between a perfluorocarbon and a liquid growth medium [14,15,95].

### 4.2. Expansion in Bioreactors

For large-scale cell expansion, several different methods have been developed, but the most common are spinner flasks for small scale uses and stirred-tank bioreactors for larger applications. Both use mechanical forces to mix the cell culture and allow the cells to grow. Putting microcarriers in the bioreactor system is a promising technique for cell expansion because it allows for a higher expansion ratio, controlled environment, and ability for the process to be scaled up [14,44,96]. The most common type of bioreactor for cell expansion on microcarriers is a stirred-tank system, but other systems exist, such as fixed-bed, fluidized-bed, airlift, or WAVE^®^ bioreactors (Figure 5E–G) [14,15,97,98,99,100]. Additionally, the properties of the system such as impeller geometry, speed, and microcarrier density should be optimized to promote cell growth and proliferation. In fact, having too high of a microcarrier density can be harmful to cell expansion because collisions between microcarrier particles can impede cell growth. A study investigating the optimal system parameters for hMSC expansion was conducted by Hewitt et al. using a spinner flask with Cytodex 3 microcarriers, made from collagen and dextran. They found that no cell damage occurred when the spinner flask operated at minimum speed, and optimal starting parameters were 3000 microcarriers per ml, 5 cells per microcarrier, and a one-day delay for cells to adhere to the microcarriers [101]. Similarly, Rafiq et al. studied the expansion of bone marrow-derived hMSCs on microcarriers and successfully demonstrated their growth in a stirred-tank 5 l bioreactor on P 102-L microcarriers, made from polystyrene, over a 12-day period. Their research supported the successful extraction of considerable amounts of cells from the microcarriers in the larger scale bioreactor system [102]. These effects of system parameters on cell viability and behavior that were observed experimentally have also been supported by computational modeling. A study by Berry et al. used computational fluid dynamics to understand the fluid flow in a stirred-tank bioreactor. This study found that there needed to be enough stirring force to support the exchange of nutrients and suspension of the microcarriers but too much stirring could generate higher shear stress, which could damage the cells or promote premature differentiation [103]. Thus, it is important to modify bioreactor characteristics for optimal cell growth and expansion.

### 4.3. Cell Expansion on Microcarriers Relevant to Cartilage TE

A number of studies have shown successful cell expansion on microcarriers with both stem cells and chondrocytes [45,92,101,105,106,107,108]. In terms of expansion of stem cells on microcarriers, hMSCs, human embryonic stem cells (hESCs), human pluripotent stem cells (hPSCs), and hADSCs are all relevant types of stem cells that have the potential for use in cartilage TE [101,102,109,110,111]. For instance, a study performed by Fan et al. found that hPSCs could survive and proliferate in a stirred-suspension bioreactor on polystyrene microcarriers coated with a peptide derived from vitronectin [106]. Phillips et al. sought to investigate the growth and attachment of hESCs on polystyrene microcarriers and compared cells harvested by collagenase dissociation, which formed aggregates, and those prepared by TrypLE^TM^ dissociation, which remained as single cells. They found that the cells quickly attached to the beads and then proliferated and that single-cell cultures expanded more than hESC aggregates [109]. Nie et al. also expanded hESC colonies on Matrigel-coated microcarriers and microcarriers seeded with a mouse embryonic fibroblast (MEF) monolayer and compared the growth of the hESCs on the microcarriers to that on tissue culture plates that were either pre-seeded with MEFs or pre-coated with Matrigel. The doubling times of the hESCs were very similar on the Matrigel-coated microcarriers and on the Matrigel-coated plates; however, the expansion rate of the hESCs on the MEF-plates was more than that on MEF-microcarriers. Importantly, the hESCs that were cultured on the microcarriers remained pluripotent, and their recovery rates after cryopreservation increased when on the microcarriers [110]. This capacity for stem cells to retain their stemness, which is their ability to differentiate and self-renew, after cell expansion is vitally important for the therapeutic potential of stem cells in regenerative approaches [112]. As a last example of stem cell expansion, Muoio et al. sought to grow hADSCs on microcarriers in serum- and xeno-free medium and found that the stem cells remained undifferentiated when grown on a novel biodegradable microcarrier [111]. Overall, these findings encourage further research since these types of stem cells can be differentiated into chondrocytes, thus allowing for application to cartilage TE.

Taking a step beyond simple cell expansion, stem cells can undergo cell expansion on microcarriers first and then be differentiated into chondrocytes while still on the microcarriers. A study by Gupta et al. used a spinner flask system for expansion of hPDCs on Cultispher S microcarriers, showing that the cells maintained their stem cell phenotype and viability for the first twelve days of culture in a growth medium. The hPDCs exhibited trilineage differentiation potential after this cell expansion, meaning that they maintained the ability to differentiate into either chondrocytes, osteoblasts, or adipocytes. However, when the cells on the microcarriers were further exposed to differentiation medium containing TGF-β and allowed to form larger clusters, they were primed to differentiate towards the chondrogenic lineage, thus demonstrating potential application to cartilage TE [113]. This shows that microcarriers in combination with certain growth factors can induce chondrogenic differentiation and chondrogenesis in situ.

Instead of stem cells, chondrocytes can also be directly expanded on microcarriers. For application to cartilage TE, it is important for chondrocytes to maintain their phenotype after cell expansion because they often undergo dedifferentiation and lose their specific phenotype during expansion that occurs ex vivo [105]. Schrobback et al. demonstrated the successful proliferation of human chondrocytes on gelatin microcarriers in a stirred-tank bioreactor, with stable cell viability for a month and most cells retaining their chondrogenic phenotypes for the first 2 weeks. Interestingly, expanded cells released from the microcarriers exhibited chondrogenic capacity in a standard pellet culture, but pellets formed with the cells still attached on the microcarriers did not [105]. In a more recent study conducted by Georgi et al., chondrocytes were expanded on Cultisphere G microcarriers in spinner flasks. The cell-laden microcarriers were then assembled into macrotissues, and co-culture of microcarriers with hMSCs along with the microcarriers with chondrocytes led to improved glycosaminoglycan deposition and chondrogenic gene expression in the macrotissues [108].

In summary, microcarriers are extremely important for advances in cartilage TE because they allow for large-scale cell expansion, which is necessary for therapies that require significant numbers of cells. Additionally, bioreactors can be used to help with the cell expansion on microcarriers by providing a controlled environment for cell growth. For application to cartilage TE, studies have shown the successful expansion of stem cells on microcarriers, which can further undergo chondrogenic differentiation, or alternatively, chondrocytes can be directly expanded on the microcarriers. Thus, research involving microcarriers is promising for cartilage TE, and further studies should investigate the long-term viability and regenerative potential of the expanded stem cells or chondrocytes so that they could be used in clinical settings.

## 5. Microspheres Forming Granular Hydrogels as 3D Tissue Engineering (TE) Constructs

Microspheres can be molded or formed together to create granular hydrogels, a relatively new application that has great potential for TE [21]. In this section, we first cover the relevant features of these materials for bioprinting, which is accomplished through the process of jamming or condensing hydrogel microparticles (HMPs) together. This gives the material important shear thinning properties [20]. We then go on to discuss MAP hydrogels, which can form TE scaffolds via crosslinking of the particles [11]. Both of these variations of granular hydrogels are promising for cartilage TE innovations.

### 5.1. Granular Hydrogels for Bioprinting Applications

**Figure 6:**
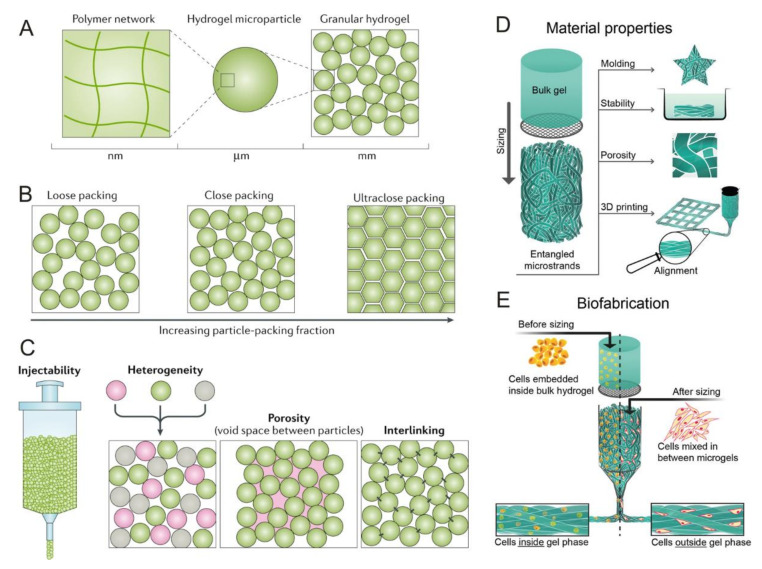
Schematics illustrating the structure and properties of granular hydrogels made from (**A**–**C**) microspheres and (**D**,**E**) entangled microstrands. (**A**) Granular hydrogels are made up of hydrogel microparticles (HMPs), which are typically made up of crosslinked polymer networks. (**B**) HMPs within the granular hydrogels can have different types of packing, including loose, close, and ultraclose. (**C**) Granular hydrogels have properties including injectability, heterogeneity (formed from mixtures of different types of HMPs), porosity between the HMPs, and ability for the HMPs to be linked together. (**D**) The process of making entangled microstrands from a bulk hydrogel involves extruding it through a grid. These microstrands can form stable and porous granular hydrogels and can be shaped by molding or 3D printing. (**E**) Bioinks can be prepared by interlaying cells in the hydrogel either before or after the microstrands are created from the bulk hydrogel. (**A**–**C**) Reproduced with permission from [115]. © 2021 Springer Nature Limited. (**D**,**E**) Reproduced under the terms of the Creative Commons CC BY License from [23]. © 2021 The Authors. Published by Wiley-VCH Verlag GmbH & Co. KGaA.

The utilization of 3D printing technology with the combination of biomaterials and cells is called bioprinting, a promising method for creating TE constructs by building up layers of material on top of each other to form a 3D structure [114]. There are various bioprinting techniques, with the most preferred deposition technique being extrusion due to its convenience, ease of application, precision, flexibility, and high levels of cell compatibility [20]. Granular hydrogels have become promising for use in extrusion-based bioprinting processes, and this can be attributed to their ability to transition between liquid and solid states via the process of jamming, which is when the particles condense together. As illustrated in Figure 6A–C, granular hydrogels are made of HMPs, which pack together to form a solid-like gel phase during jamming and then can undergo unjamming to form a viscous fluid when under shear stress [20,115]. The use of the HMPs in the granular hydrogels gives the material good shear-thinning and self-healing properties, making it ideal for extrusion-based bioprinting with the possibility to be used either as a bioink or as a supporting bath [20]. Zhang et al. recently utilized jammed HMPs made from Carbomer 980 as a supporting bath and 3D printed an ECM solution made from bovine Type I collagen into the void spaces between the HMPs in order to support the growth of cells. The jammed HMP material could be used for bioprinting of collagen 3D structures with minimal intermixing of the collagen and HMP materials, thus allowing for potential use in TE [116]. In a different approach, bioprinted granular hydrogels were shown to be able to support chondrogenic differentiation of hMSCs. The cells were encapsulated into HMPs made from oxidized and methacrylated alginate, which allowed for dual crosslinking to form the HMPs and to stabilize the printed structures made from them. The cell-laden HMPs were successfully bioprinted into 3D structures. Furthermore, when added to a cell culture medium containing TGF-β1 for 4 weeks, the hMSCs in the HMPs were able to differentiate to a chondrogenic phenotype. Additionally, the researchers experimented with cryopreservation of the cells while encapsulated in the HMPs and concluded that the cryopreservation did not significantly affect the chondrogenic differentiation capability of the hMSCs. Cryopreservation is useful for the storage of cells and tissues, and thus this study takes the first steps to show translational potential for this TE approach [117].

Alternatively, instead of using spherical HMPs, another relatively new method is to use elongated microparticles, called microstrands, as shown by Kessel et al. This study used entangled microstrands (Figure 6D,E) for bioprinting chondrocytes with application to cartilage TE. Microstrands offer certain advantages over traditional spherical microparticles such as enhanced interaction with the environment, better stability, and the ability to align when passing through the nozzle of the bioprinting device. They are created by forcing a bulk hydrogel through a sieve with openings of 40–100 µm to create individual strands, which then entangle with each other in a random fashion. Secondary crosslinking to strengthen the structure occurs via exposure to ultraviolet light after the strands have been formed. In this experiment, discs composed of hyaluronan-methacrylate were bioprinted through pneumatic extrusion of the entangled microstrands, and bovine chondrocyte cells were cultured on the scaffold in vitro over six weeks. Although the chondrocytes started losing their typical rounded morphology by the third week, they maintained 90% viability for the entire 6 weeks and produced relevant ECM such as glycosaminoglycans and Type II collagen [23]. Thus, entangled microstrands have the potential for cartilage TE and should be investigated in further studies.

### 5.2. Microporous Annealed Particle (MAP) Hydrogels

**Figure 7:**
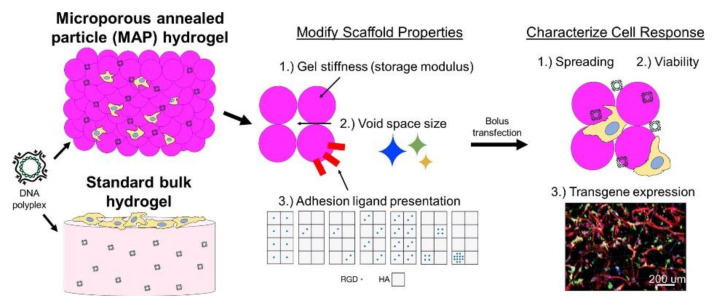
Schematics showing microporous annealed particle (MAP) hydrogels when compared to standard bulk hydrogels. Properties of the MAP hydrogels, including hydrogel stiffness, void space size, and cell adhesion ligand presentation, can be examined to characterize their effects on cellular responses, such as cell spreading and viability as well as transgene expression. Reproduced with permission from [118]. © 2021 Elsevier.

Besides jamming HMPs together for 3D bioprinting applications, another method to prepare TE scaffolds from microspheres is by creating MAP hydrogels (Figure 7). This approach crosslinks spherical HMPs together in situ to form a scaffold with gaps between the packed microparticles in which cells can grow [11,118]. MAP scaffolds have several advantages for TE both in vitro and in vivo, due to their porous structure, allowing for better cell infiltration and growth in the scaffold [11,118]. Traditionally, for creating microporous scaffolds, the majority of studies have followed a top-down approach by taking a bulk material and degrading sections of it to create pores. However, the synthesis of granular hydrogels and MAP scaffolds follow a bottom-up approach where microparticles are formed together into a porous material through either jamming or crosslinking. This approach can be useful to create a complex environment for cell growth with heterogeneous particles varying in size and surface chemistry. For example, variation in microparticle size was used to control the morphology (spread vs. round) of hMSCs grown in MAP scaffolds formed from PEG microparticles functionalized with the cell adhesion ligand, arginine-glycine-aspartic acid (RGD) [119]. Overall, for MAP scaffolds, the biochemical and mechanical properties, such as heterogeneity, porosity, and interlinking, can be easily tuned to adjust the rates of cell adhesion and proliferation, allowing for materials that can be adapted for different environments [11,118,119].

**Figure 8:**
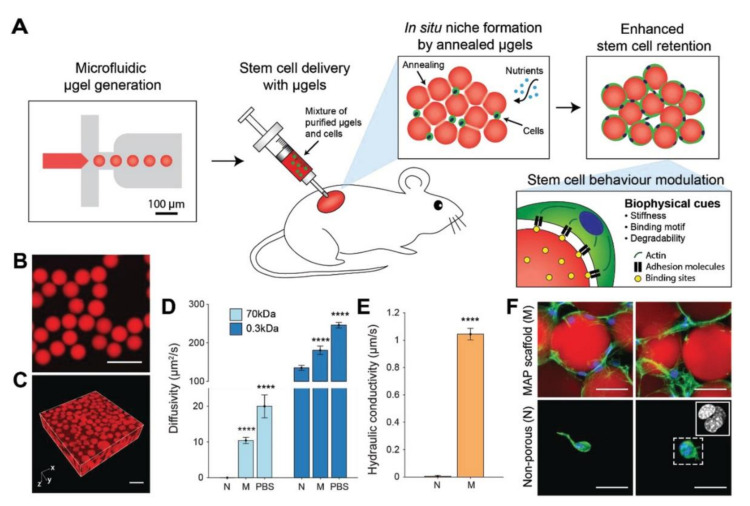
(**A**) Schematics showing how monodisperse hydrogel microparticles (HMPs) are generated and allow for the controlled creation of a pore network within microporous annealed particle (MAP) hydrogels, ultimately promoting cell movement and the transport of oxygen and nutrients. The function of the transplanted stem cells is enhanced by the presence of the hydrogel. (**B**,**C**) Micrographs of the fluorescent HMPs. (**D**,**E**) Materials characterization. (**F**) Micrographs of cells on the hydrogel scaffolds. Reproduced with permission from [11]. © 2021 Wiley-VCH Verlag GmbH & Co. KGaA.

As MSCs can be induced to differentiate into chondrocytes, their incorporation into granular hydrogels is important in order to encourage heightened success for cell growth and viability [11,119]. Koh et al. found that the successful and localized delivery of stem cells for TE occurred when using injectable scaffolds through the fabrication of MAP scaffolds made via in situ crosslinking of monodisperse HMPs primarily made of PEG (Figure 8). These HMPs were either degradable or non-degradable and were functionalized with unique, differing amounts of the cell adhesion ligand, RGD. When MSCs were co-injected subcutaneously in mice with the MAP scaffolds formed in situ, MSCs showed enhanced migration, maintenance, adhesion, and regeneration abilities in vivo compared to when using chemically similar nonporous hydrogels. The formation of the spherical HMPs led to increased nutrient transport, enhanced diffusivity, cell connectedness, and cellular migration due to the interconnected microscale void spaces it created. Furthermore, in a mouse subcutaneous implantation model, these hydrogels led to MSC half-lives up to three times more than that of MSCs in chemically identical nonporous hydrogels. The flexibility of varying the material properties helped encourage stem cell proliferation and integration with tissues surrounding the damaged or inflamed area via vascularization [11]. Through the utilization of a similar PEG-based MAP scaffold, Caldwell et al. found that MSCs had around 95% viability after a 96-h culture period in vitro; the scaffolds allowed for cell proliferation and interaction, high tunability, and flexibility regardless of tissue environment. These findings suggest that MAP scaffolds warrant further research as they can aid in better promoting cellular activities for in vitro and in vivo tissue regeneration purposes [119].

Evidently, the conglomeration of microspheres into granular hydrogels has the potential to revolutionize the TE space, although it is a relatively new area of research. While there are many potential benefits to incorporating granular hydrogels into cartilage TE processes, more research is needed as there has not been sufficient experimentation in terms of using these materials specifically for cartilage TE.

## 6. Conclusions

Although TE approaches to cartilage repair are still in development, their potential to revolutionize the medical space while improving the lives of millions of humans warrants further research. However, uncertainties surrounding the use of various cell types and scaffolds in TE have dominated discussions. We believe that the utilization of biomaterial technologies based on microparticles, such as the microspheres, microcarriers, and granular hydrogels described herein, will serve to encourage and expedite successful development processes. Microspheres can be used as delivery systems to administer drugs that promote cartilage regeneration, including growth factors, corticosteroids, and anti-inflammatories. The targeted and controlled release of drugs or proteins allowed with microspheres make them an ideal treatment for cartilage degeneration and pain as well as for use in TE. Microcarrier technology can be applied for TE by allowing large-scale cell expansion, especially when used in a bioreactor system, and the proliferation of MSCs on microcarriers has direct applications to cartilage TE and cell-based therapies. Additionally, microparticles can be used to form granular hydrogels either through jamming or crosslinking them together. The hydrogels formed by jamming can create scaffolds that have excellent shear thinning properties for 3D bioprinting, making this technology an encouraging methodology for creating TE scaffolds. Through the use of MAP scaffolds, stem cells have shown enhanced cellular activities both in vivo and in vitro, and thus these materials can potentially be used in cartilage TE.

Future research should thus be aimed towards further evaluating and investigating the potential incorporation of microspheres and microcarriers specifically for cartilage TE. Expansion of stem cells on microcarriers in bioreactors have shown successful chondrogenic differentiation capabilities, but their long-term viability and maintenance of phenotype should be studied more in-depth in future studies. Some other areas of research to investigate include using entangled microstrands as an alternative to microspheres when forming jammed hydrogel scaffolds as well as evaluating MAP scaffolds using in vivo models of cartilage degeneration. After successful preclinical animal studies, microspheres in conjunction with different drugs for cartilage regeneration or pain relief can continue moving into clinical trials. Additionally, cryopreservation studies have significant application to long-term storage of TE materials, and the maintenance of viability and potential for cell expansion of stem cells on microcarriers after cryopreservation should be further investigated.

## Data Availability

This review article did not report any new data.

## Abbreviations

ACI	Autologous chondrocyte implantation
BMP	Bone morphogenetic protein
BMSC	Bone marrow-derived mesenchymal stem cell
COX-2	Cyclo-oxygenase-2
CXB	Celecoxib
dCM	Decellularized cartilage matrix
DLMS	Dexamethasone-loaded microspheres
ECM	Extracellular matrix
hADSC	Human adipose-derived stem cell
hESC	Human embryonic stem cell
HMP	Hydrogel microparticle
hMSC	Human mesenchymal stem cell
hPDC	Human periosteum-derived cell
hPSC	Human pluripotent stem cell
IL	Interleukin
INJ	Injected group
IVD	Intervertebral disc
MAP	Microporous annealed particle
MEF	Mouse embryonic fibroblast
MSC	Mesenchymal stem cell
NP	Nucleus pulposus
NSAID	Non-steroidal anti-inflammatory drug
OA	Osteoarthritis
OARSI	Osteoarthritis Research Society International
pDNA	Plasmid deoxyribonucleic acid
PEA	Polyesteramide
PLA	Polylactic acid
PLGA	Polylactic-co-glycolic acid
RGD	Arginine-glycine-aspartic acid
TAA	Triamcinolone acetonide
TE	Tissue engineering
TGF	Transforming growth factor
US	United States
3D	Three-dimensional
µCT	Microcomputed tomography
